# Interventions to Prevent Intimate Partner Violence: A Systematic Review and Meta-Analysis

**DOI:** 10.1177/10778012231183660

**Published:** 2023-07-20

**Authors:** Ema Alsina, Joyce L. Browne, Desi Gielkens, Maaike A. J. Noorman, John B.F. de Wit

**Affiliations:** 1 168086 Julius Center for Health Sciences and Primary Care, University Medical Center Utrecht, Utrecht, The Netherlands; 2 Department of Interdisciplinary Social Science, Utrecht University, Utrecht, The Netherlands

**Keywords:** intimate partner violence, sexual violence, preventive intervention, systematic review, meta-analysis

## Abstract

Intimate partner violence (IPV) remains a global health and human rights problem. This systematic review assesses the effects of preventive interventions on the occurrence of IPV experience or perpetration. Twenty-six studies published between January 1, 2008 and March 31, 2022 were included, contributing 91 effect sizes. Multilevel meta-analysis showed a protective pooled effect (risk ratio = 0.85, 95% CI [0.77, 0.99]). Interventions (also) including men were more effective than interventions for women only. No other moderators were found. Findings underscore that various IPV prevention interventions are now available that can improve the health and rights of women in diverse settings.

## Introduction

The World Health Organization defines intimate partner violence (IPV) as *“behaviour within an intimate relationship that causes physical, sexual or psychological harm, including acts of physical aggression, sexual coercion, psychological abuse and controlling behaviours. This definition covers violence by both current and former spouses and partners”* (World Health Organization/London School of Hygiene and Tropical Medicine, 2010). IPV occurs everywhere in the world, albeit there is variation in regional prevalence and severity (García-Moreno et al., 2015; Starrs et al., 2018). IPV is a major public health and human rights issue, and 2018 estimates based on data from 161 countries and areas show that 27% of ever-partnered women aged 15–49 years globally experienced physical and/or sexual IPV in their lifetime, with 13% experiencing IPV in the past year (Sardinha et al., 2022). Women in low- and middle-income countries are more likely than women in high-income countries to have experienced IPV, in particular in the past year (Sardinha et al., 2022). IPV is associated with a variety of adverse health outcomes for women and their children (Campbell, 2002), including death, physical injury, mental health problems, alcohol and substance abuse, HIV infection, and adverse pregnancy outcomes (Ellsberg et al., 2008; Janssen et al., 2003; Kouyoumdjian et al., 2013). The elimination of violence against women and girls, including IPV, is set out in Sustainable Development Goal 5.2 (United Nations, 2015).

Preventive interventions have the potential to reduce the occurrence of IPV, in particular if they are guided by a theory of change attuned to the local context that addresses the multiple factors associated with IPV risk (cf. Jewkes et al., 2021). Alcohol consumption, witnessing or experiencing abuse as a child and cultural normativity of IPV have been found to increase IPV risk, while women with higher socio-economic status, higher education and in formal marriage are less likely to experience IPV (Abramsky et al., 2011; World Health Organization/London School of Hygiene and Tropical Medicine, 2010). Interventions intended to prevent the occurrence of IPV may hence focus on changing beliefs about gender norms, avoiding childhood exposure to violence, reducing harmful alcohol use and increasing economic empowerment (Heise, 2011). Furthermore, interventions primarily focused on other health and social issues that increase the risk of IPV, such as HIV/STI, alcohol/drug abuse and economic interventions, may also have beneficial effects on experiences and perpetration of IPV (Abramsky et al., 2011; Anderson et al., 2013; Dunkle & Decker, 2013; García-Moreno et al., 2015; Hines, 2007; Jewkes, 2002; World Health Organization/London School of Hygiene and Tropical Medicine, 2010).

We undertook a systematic review of existing research to contribute to further strengthening the evidence base regarding effective interventions to prevent the occurrence of any experience or perpetration of IPV, including the initial, ongoing or re-occurrence of IPV. Our evidence synthesis contributes in three ways to the existing literature. Firstly, our evidence synthesis builds on the seminal World Health Organization/London School of Hygiene and Tropical Medicine (2010) review and reviews research that has been published since (i.e., as of 2008). While the World Health Organization/London School of Hygiene and Tropical Medicine (2010) review found that only one intervention strategy was demonstrated to be effective, research on IPV prevention has proliferated in recent years and several approaches have since found to be effective (Jewkes et al., 2021).

Secondly, systematic reviews of research assessing the effects of IPV prevention interventions abound, but these tend to have a specific focus, including on particular regions, such as sub-Saharan Africa (e.g., Cork et al., 2020), particular population groups, such as women around the time of pregnancy (e.g., Van Parys et al., 2014), men who use substances (e.g., Stephens-Lewis et al., 2021) or adolescents and young adults (e.g., Piolanti & Foran, 2022). Evidence syntheses have also been published for specific types of interventions, such as screening women in healthcare settings (e.g., O'Doherty et al., 2015), economic empowerment (e.g., Eggers Del Campo & Steinert, 2020) or combined IPV and HIV prevention (e.g., Marshall et al., 2018). We extend existing systematic reviews of IPV intervention studies by applying a broad rather than specific focus that enables the comparison of effects of different types of interventions, for different population groups and in different regions. We also examine whether the types of interventions that have been tested to prevent IPV differ between countries and population groups.

Thirdly, we further extend current evidence syntheses by undertaking a meta-analysis to provide a quantitative estimate of the effect of preventive interventions on the occurrence of any experience or perpetration of IPV. Most evidence syntheses to date consist of systematic reviews that provide a narrative summary of findings. Extending a systematic review, which can provide a count of the number of studies that did or did not find an effect, a meta-analysis produces a numerical estimate of the strength of the effect across studies. By pooling and weighting data from multiple studies, a meta-analysis provides a more robust estimate of intervention effects than single studies. Furthermore, whereas a systematic review can provide indications of similarities and differences between interventions that are or are not effective, the use of meta-regression techniques enables testing of the extent to which characteristics of the intervention and the research are associated with intervention effects. Meta-analysis is hence considered to produce the highest level of evidence to guide healthcare decisions (Guyatt et al., 1995).

Fourthly, to the best of our knowledge, our meta-analysis uniquely employs a multilevel approach to estimating the pooled intervention effect. Studies typically provide results on multiple outcomes, and when the nonindependence of such nested effects is ignored, conclusions based on conventional meta-analysis procedures are likely incorrect (Cheung, 2019). Also, established strategies to circumvent problems due to the interdependence of effects, notably averaging effects within a study or selecting one effect per study, result in missed opportunities to use all available data (Cheung, 2019). A multilevel meta-analysis is an underutilized approach to effectively handle the nonindependence of effects and make use of all available data (Assink & Wibbelink, 2016).

## Method

### Protocol Registration

The protocol of this systematic review and meta-analysis was registered with PROSPERO (CRD42018073921). When undertaking the revision of this manuscript, several changes to the protocol were implemented to clarify and strengthen the evidence synthesis, as indicated below. Reporting adhered to the PRISMA guidelines (see Page et al., 2021).

### Databases and Search

The literature search for the systematic review was conducted in three steps. As per study protocol, we initially undertook a search of PubMed and Cochrane databases to identify eligible studies published between January 1, 2008 and December 31, 2017, building on a World Health Organization report that summarized evidence from research on interventions to prevent the occurrence of intimate partner and sexual violence up to 2008 (World Health Organization/London School of Hygiene and Tropical Medicine, 2010). In the second step, undertaken as part of the revision of the manuscript, we broadened the systematic review to include a literature search of four major databases of scholarly literature in fields relevant to the public health focus of this evidence synthesis: PubMed, CINAHL, Web of Science, and Scopus. In the third step, also encompassed in the manuscript revision, we extended the period of publication to identify eligible studies published a untill March 31, 2022. To identify any potential studies missed, we screened the reference lists of included papers and, where possible, screened papers that cited included papers. Furthermore, we screened the reference list of (systematic) reviews of research regarding the effectsof interventions to prevent IPV (references are available from the corresponding author).

As also specified in the protocol, we initially used the combination of the following search terms to identify potentially eligible studies: “intimate partner violence,” “IPV,” “sexual violence,” or “domestic violence” in the title or abstract, as well as “intervention,” “reduce,” “prevent,” or “trial.” For the broadened and extended literature searches, we adjusted the search terms and their combination, using the following Boolean search string: (“intimate partner violence” OR IPV OR “sexual violence” OR “domestic violence” OR “dating violence” OR “gender-based violence” OR GBV) AND (intervention OR program) AND (trial OR study) AND (random* OR “quasi experiment*”) for title/abstract/keyword searches. The specification of the search string was amended, as needed, to meet the technical requirements of the specific database searched, while ensuring the substantive correspondence between the searches of different databases.

### Inclusion/exclusion Criteria

For this evidence synthesis, we only considered IPV perpetrated by men against women, which constitutes the vast majority of IPV (e.g.,World Health Organization/London School of Hygiene and Tropical Medicine, 2010). According to the protocol, studies were eligible for inclusion if they met the following criteria: (a) published between January 1, 2008 and December 31, 2017, which we extended to March 31, 2022 (i.e., the date when the broadened and extended literature search was completed), (b) included adult study participants (specified as participant age of inclusion of 15 years or over), (c) reported on the occurrence or re-occurrence of at least one form of IPV, (d) longitudinal assessment of outcomes (i.e., the inclusion of pre- and postintervention assessments), and (e) reported findings as odds ratios or risk ratios (RRs) with confidence intervals, or as regression coefficients with standard errors (SEs). We additionally specified that studies were eligible when (f) they had a (cluster) randomized controlled trial design or a quasi-experimental design with the robust matching of comparison groups, and (g) reported a sufficiently detailed sample size analysis providing evidence of sufficient power to identify any significant intervention effects on the IPV outcomes. Study outcomes were ineligible if they pertained to (a) indicators of frequency of IPV experiences that could not be transformed into rates of any (re-)occurrence (yes/no), (b) survivor (mental) health and well-being outcomes, or (c) attitudes or intentions related to IPV or gender inequality. We also excluded (d) outcomes related to paying partner violence against female sex workers.

### Study Selection

The team jointly developed and implemented the literature search strategy. In the first step of the evidence synthesis, the literature search and title screening were conducted by EA. The abstract screening was subsequently performed independently by EA and DG, using the Covidence systematic review software (Veritas Health Innovation, www.covidence.org); disagreements were resolved by discussion. The revised search strategies for the broadened and extended literature reviews were developed by JdW, in consultation with JB. JdW undertook title and abstract screening, and MN independently evaluated a random sample of 100 records to verify title screening and of 50 records to verify abstract screening. No disagreements were noted. When multiple publications were found that reported on the same intervention evaluation, the most recent and complete study was retained.

### Data Extraction

A data extraction sheet was initially developed by EA, in collaboration with the other researchers. For the second and third steps of the evidence synthesis, the data extraction sheet was amended by JdW, who undertook data extraction for the broadened and extended literature reviews. We extracted publication identifiers (i.e., authors, title, year of publication), research characteristics (year of study start, study design, country, location, setting, number of participants, participants’ gender), characteristics of intervention and control or comparison conditions (i.e., type of intervention and control or comparison conditions, theoretical foundation), characteristics of outcomes (i.e., type of IPV outcome, outcome measure used, period of assessment, period of follow-up), and details of data analysis (i.e., intention-to-treat or per protocol, adjustments) and intervention effects (including confidence intervals or SEs) for each outcome.

### Risk of Bias

For the studies included following the initial literature search, the risk of bias (RoB) was independently assessed by EM and DG, using the Cochrane revised RoB tool (RoB 2) for RCTs (Sterne et al., 2019) and its supplement for cluster RCTs (for details see www.riskofbias.info). For the studies identified through the extended and broadened literature searches, the RoB was assessed by JdW, also using the RoB 2 tool. MN independently assessed a random sample of half of the included studies; discrepancies were resolved through discussion and any changes in scoring were implemented for all included studies. The ROB 2 tool encompasses assessments of possible bias in five domains: randomization process, deviations from the intended interventions, missing outcome data, measurement of the outcome, and selection of the reported result. The RoB was considered a potential moderator of effect size; it did not influence study inclusion in the meta-analysis.

### Meta-Analysis

Data analysis was undertaken in R, using the R Studio interface (see www.rstudio.com). Data analysis and reporting benefited from the guidance provided by Harrer et al. (2021). Random effects modeling was chosen for the meta-analysis, as this method does not assume similar underlying effect sizes for each study, allowing for possible heterogeneity of intervention effects (Hedges & Vevea, 1998). For ease of interpretation, we used RRs and related SEs to assess the size of intervention effects. Odd Ratios were converted as specified by Zhang and Yu (1998). No other effect measures were reported for included studies. All effect sizes were log-transformed for the purpose of the analyses. For ease of interpretation, we report the untransformed Relative Risk.

We used the metafor package (Viechtbauer, 2010) to produce a forest plot of included intervention effects and undertake multilevel meta-analysis accounting for the interdependence of multiple intervention effects reported by studies. The restricted maximum likelihood estimator (Viechtbauer, 2005) was used to calculate heterogeneity variance. We used the dmetar package (Harrer et al., 2019) to calculate the multilevel I^2^ statistic (Cheung, 2014) that assesses the extent of heterogeneity due to within- and between-study differences. To assess if the multilevel model provided a better fit to the data than a simple random effects model, we used the analysis of variance function in the dmetar package to compare the simple and multilevel models. We undertook meta-regression analyses using the metafor package to assess differences in effect size associated with main study characteristics.

## Results

### Study Selection

**Figure 1.**
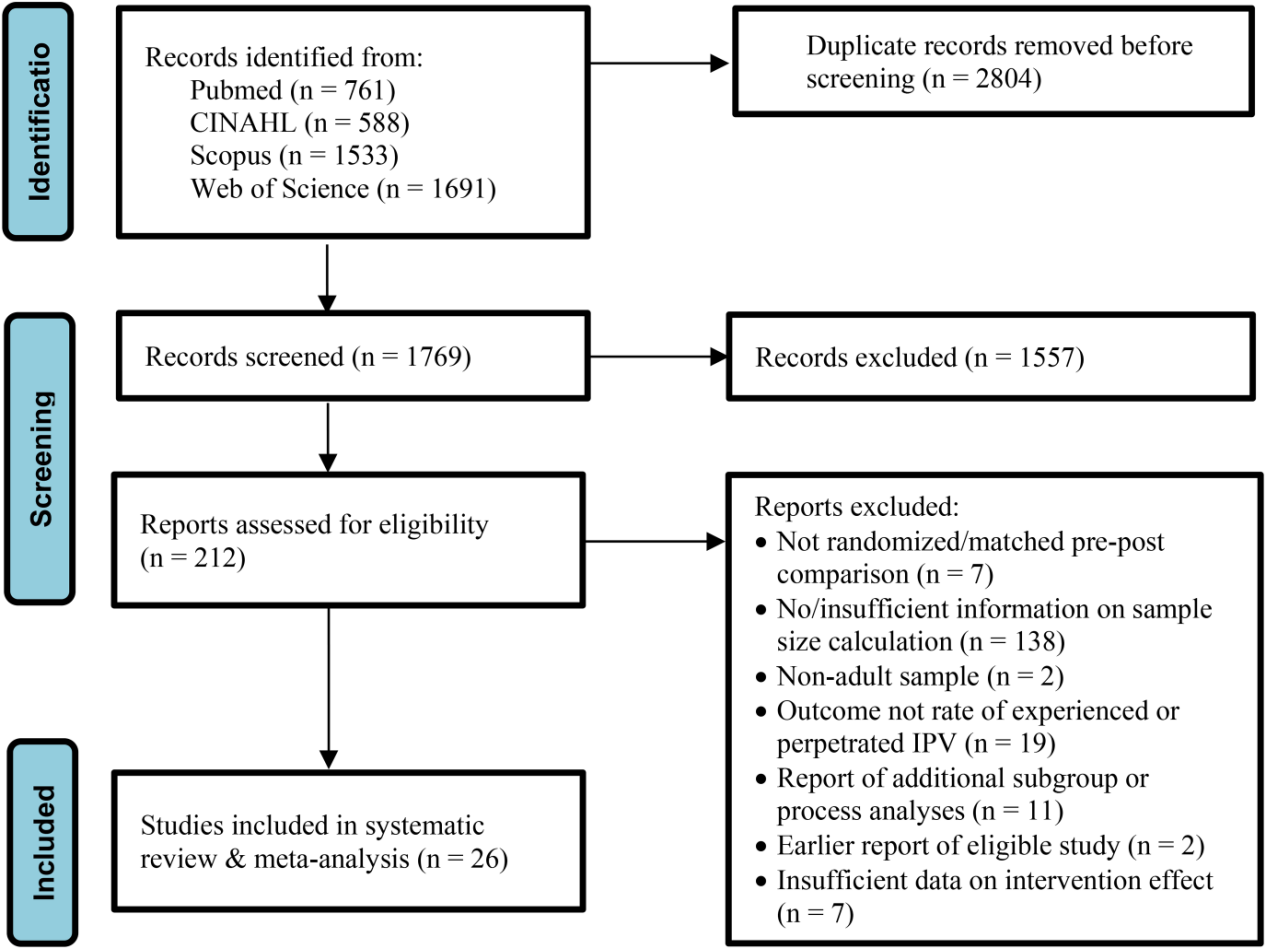
Selection of Eligible Studies (PRISMA Flow Chart Adapted From Page et al., 2021).

The flow of the process to identify and select studies for inclusion is shown in Figure 1. We identified 4,573 records across four databases of scientific publications. No additional studies that met inclusion criteria were identified through citation screening. Of the 1,769 unique records remaining after the removal of duplicates, 1,557 were excluded based on title/abstract screening. The full texts of the remaining 212 articles were assessed and 26 studies were found to meet eligibility criteria. We applied an ordered assessment of eligibility, starting with an appraisal of the study design, details regarding sample size calculation, sample age, and outcome measures. We also assessed if the articles only reported additional subgroup or processes analyses, were earlier reports of eligible studies and provided sufficient data on intervention effects. Were relevant and available, we accessed published study protocols or other study publications to identify the information required to appraise eligibility. Note that studies that were excluded based on an earlier assessment criterion could also have been ineligible because of a later criterion (e.g., studies of which articles did not include sufficient details on sample size calculation may also have been ineligible because these did not report on, for instance, eligible samples or outcomes).

### Study Characteristics

An overview of the characteristics of the 26 included studies assessing the effect of interventions to prevent the occurrence of any experience or perpetration of IPV is shown in Table 1. Most studies were conducted in low- and middle-income countries, particularly in sub-Saharan Africa (14 studies). Interventions were predominantly implemented in urban areas and in community settings. Most studies tested interventions for women only, with only two studies including (an intervention arm for) men only; 10 studies tested interventions (in an intervention arm for) men and women, including but not limited to couples. About a third of intervention studies only included women who had previously experienced IPV or were at high risk of IPV; most studies included any women who currently or recently had a male partner. Study sample sizes ranged from 189 to 11,448.

**Table 1.** Overview of Characteristics of Included Studies.

Citation	Country, location, setting, start year	Design	Participants, number	Intervention	Control	Purpose, theory	Outcome(s), period, follow-up	Outcome measure, assessment
Abramsky et al. (2016)	Uganda, Kampala, community sites, 2007	cRCT	Community members (18–49 years old), 2,532	Community mobilization	No intervention	Prevention of VAW, Ecological Model of Violence, Stages of Change Theory	IPV experienced by women (physical, sexual, physical and/or sexual, emotional), 12 m, 48 m	WHO VAW scale, participant interview
Decker et al. (2020)	Kenya, Nairobi, informal settlements, 2018	RCT	Women (18–35 years old) at risk of or experiencing IPV, 352	Multicomponent app	Standard IPV referrals	Prevention of VAW, Decision Science, Social Cognitive Theory, empowerment, trauma-informed care	IPV experienced by women (physical, sexual, physical and sexual), 3 m, 3 m	CTS2, participant survey with assistant
Doyle et al. (2018)	Rwanda, rural districts, communities, 2015	RCT	Expectant/current fathers (21–35 years old) and their female partners, 1,199	Gender transformative couples’ intervention	Community activities and campaigns only	Reproductive and maternal health, theories of gender and masculinities	IPV experienced by women (physical, sexual), 12 m, 21 m	WHO VAW scale, participant interview
Dunkle et al. (2020)	Rwanda, rural districts, savings and loans associations, 2015	cRCT	Couples (18–49 years old), 3,311	Couples’ curriculum, community outreach, creating an enabling environment, and support for victims	Village savings and loans association only	Prevention of VAW, not identified	IPV experienced by women (physical, sexual, physical and/or sexual), IPV perpetrated by men (physical, sexual, physical and/or sexual), 12 m, 24 m	WHO VAW scale, participant interview
Gibbs et al. (2020)	Afghanistan, urban and periurban locations, communities, 2016	RCT	Married women (18–49 years old), 933	Combined economic and social empowerment program	No intervention	Prevention of VAW, not identified	IPV experienced by women (physical), 12 m, 22 m	WHO VAW scale, participant interview
Gibbs et al. (2020)	South Africa, eThekwini Municipality, informal settlements, 2015	cRCT	Young people (18–30 years old) not in formal employment or education, 1322	Participatory group training and livelihood strengthening	Waitlist	HIV prevention, adult learning theories	IPV experienced by women (physical, sexual), IPV perpetrated by men (physical, sexual), 12 m, 24 m	WHO VAW scale, participant survey
Gilbert et al. (2016)	USA, New York City, community corrections sites, 2009	RCT	Substance-using women (18 years or older) in community correction, 306	HIV and IPV prevention group sessions with or without computerized modules	Wellness promotion	HIV prevention, Social Cognitive Theory, Empowerment Theory	IPV experienced by women (physical, sexual), 6 m, 12 m	CTS2, participant assessment
Gupta et al. (2013)	Cote d'Ivoire, rural locations, villages, 2010	RCT	Women (18 years or older) and male partners, 934	Group savings plus gender dialog groups	Group savings alone	Prevention of VAW, Stages of Change Theory	IPV experienced by women (physical, sexual, physical and/or sexual), 12 m, 12 m	WHO VAW scale, participant interview
Gupta et al. (2017)	Mexico, Mexico City, public health clinics, 2013	cRCT	Women (18–44 years old) with recent IPV experiences, 717	IPV screening, referral, health/safety risk assessments and 3-months booster	Screening and referral card only	Prevention of VAW, not identified	IPV experienced by women (physical, sexual, physical and/or sexual), 12 m, 15 m	WHO VAW scale, participant survey
Harvey et al. (2021)	Tanzania, Mwanza City, newly formed neighborhood groups, 2015	cRCT	Women (20–50 years old) in neighborhoods, 1265	Gender transformative intervention	Waitlist	Prevention of VAW, not identified	IPV experienced by women (physical, sexual, emotional), 12 m, 24 m	WHO VAW scale, participant interview
Javalkar et al. (2019)	India, Karnataka State, villages, 2014	cRCT	Female sex workers (18 years or older) with a current or recent (last 6 months) intimate partner, 809	Multilevel intervention for sex workers, their intimate partners, and communities	Standard HIV programming	HIV prevention, not identified	IPV experienced by women (physical and/or sexual), 6 m, 24 m	WHO VAW scale, participant interview
Kapiga et al. (2019)	Tanzania, Mwanza City, microfinance loan groups, 2014	cRCT	Women in microfinance scheme (average age 39–40 years old), 1049	Violence prevention intervention	Microfinance scheme only	HIV prevention, not identified	IPV experienced by women (physical, sexual, physical and/or sexual, emotional), 12 m, 24 m	WHO VAW scale, participant survey
Kiely et al. (2011)	USA, Washington DC, prenatal care sites, 2001	RCT	African American women (18 years or older) attending prenatal care, 1,044	Individual-tailored cognitive-behavioral counseling	Usual care	Reproductive and maternal health, Empowerment Theory	IPV experienced by women (physical, sexual), 9 m, 9 m	Abuse Assessment Screen, participant interview
Koziol-Mclain et al. (2010)	New Zealand, North Island, emergency department, 2007	RCT	English-speaking women (16 years or older) attending emergency department, 344	IPV screening, unacceptability of violence messaging, risk assessment, and referral	Usual care	Prevention of VAW, Empowerment Theory	IPV experienced by women (any), 3 m, 3 m	Composite Abuse Scale, participant interview
MacMillan et al. (2009)	Canada, Ontario, emergency departments, family practices, and obstetrics/gynecology clinics, 2005	RCT	Women (18–64 years old) in health settings screened positive for IPV, 6,743	IPV screening and communication of positive result to clinician plus information card	Information card only	Prevention of VAW, not identified	IPV experienced by women (any), 6 m, 18 m	Composite Abuse Scale
Maman et al. (2020)	Tanzania, Dar-es-Salaam, social networks, 2014	cRCT	Young men (15 years or older) in “camps,” 1,258	Microfinance and peer health leadership intervention	Waitlist	HIV prevention, not identified	IPV perpetrated by men (physical and/or sexual), 12 m, 30 m	WHO VAW scale, computer-assisted personal interviews
Miller et al. (2016)	USA, Western Pennsylvania, family planning clinics, 2011	cRCT	English- or Spanish-speaking women (16–29 years old), 2,926	Education and counseling	Usual care	Prevention of VAW, Empowerment Theory	IPV experienced by women (physical and/or sexual), 3 m, 12 m	No existing instrument identified, audio computer-assisted self-interview
Murray et al. (2020)	Zambia, Lusaka, compounds, 2016	RCT	Couples of women (18 years or older) reporting IPV and their alcohol-abusing male partners, 248	Multiproblem group-based cognitive behavioral therapy	Usual care plus safety checks	Prevention of hazardous alcohol use in male partners, family therapy, relapse prevention	IPV experienced by women (physical, sexual), IPV perpetrated by men (physical, sexual), 12 m, 24 m	Severity of Violence Against Women Scale, audio computer-assisted self-interview
Rhodes et al. (2015)	USA, Philadelphia, emergency department, 2011	RCT	Women (18–64 years old) who exceeded sex-specific safe drinking limits, 600	Brief motivational intervention	No intervention	Prevention of VAW, Motivational Interviewing, Empowerment Theory	IPB experienced by women (any), 1w, 3m	CTS2, participant interview
Salazar et al. (2014)	USA, southeastern United States, university campus, 2010	RCT	Male hetero- or bisexual undergraduate students (18–24 years old), 743	Web-based bystander intervention	Web-based general health promotion program	Prevention of VAW, Social Cognitive Theory, Social Norms Theory	IPV perpetrated by men (any), 6 m, 6 m	CTS2, participant survey
Settergren et al. (2018)	Tanzania, Mbeya Region, health facilities and surrounding communities, 2012	cRCT	Women (15–49 years old) sampled from households, 1299	Comprehensive health facility- and community-based program	Usual care	HIV prevention, Social Ecological Model	IPV experienced by women (physical, sexual, emotional, any), 12 m, 28 m	No existing instrument identified, participant interview
Sharma et al. (2020)	Ethiopia, rural locations, villages, 2014	cRCT	Women (18–49 years old) and their male partners, 6770	Gender-transformative, participatory intervention (women only, men only and couples conditions) and conflict resolution	Short IPV education session	HIV prevention, not identified	IPV experienced by women (physical, sexual, physical and/or sexual, emotional), IPV perpetrated by men (physical, sexual, physical and/or sexual, emotional), 12 m, 24 m	WHO VAW scale, participant survey with assistant
Taft et al. (2011)	Australia, Melbourne, primary care clinics, 2006	cRCT	Pregnant or recent mothers (16 years or older) experiencing or at risk of IPV, 215	Nonprofessional mentor support	Usual care	Prevention of VAW, not identified	IPV experienced by women (any), 12 m, 12 m	Composite Abuse Scale, participant assessment
Vaillant et al. (2020)	Democratic Republic of the Congo, North and South Kivu, communities, 2016	cRCT	Adult men (18 years or older) and their female partners, 1,607	Group-based discussions to transform gender relations in communities	Group sessions on alternative topics	Prevention of VAW, not identified	IPV experienced by women (physical, sexual, physical and/or sexual, emotional, any), 12 m, 12 m	WHO VAW scale, computer-assisted personal interviews
Van Parys et al. (2017)	Belgium, Flanders, hospital antenatal care clinics, 2010	RCT	Women disclosing IPV (average age 27 years old), 189	IPV screening and referral card	Screening for IPV and thank you card	Prevention of VAW, not identified	IPV experienced by women (any), 6 m, 10 m	CTS2, participant telephone interview
Wagman et al. (2015)	Uganda, Rakai, community cohort study, 2005	cRCT	Women and men (15–49 years old) in community settings, 11,448	Standard of care HIV services plus community IPV mobilization, IPV screening and brief safe HIV disclosure and risk reduction intervention	Standard of care HIV services	HIV prevention, Stages of Change Theory	IPV experienced by women (physical, sexual, emotional), IPV perpetrated by men (physical, sexual, emotional), 12 m, 35 m	CTS2, participant interview

*Note.* VAW = violence against women.

We distinguished five types of IPV prevention interventions: individual support/counseling, small-group counseling, economic empowerment, community mobilization, and IPV screening and referral. Individual support/counseling interventions included single (two studies) or multisession (two studies) programs delivered by trained counselors or health care providers, weekly home visits to pregnant or recent mothers by trained mothers (one study), as well as a mobile phone app (one study). Small-group counseling interventions were delivered by trained (gender-matched) facilitators, with numbers of sessions ranging from two to 21. Sessions typically focused on strengthening motivation and skills (also referred to as empowerment), with one study including the computerized delivery of (additional) materials. Economic interventions consisted of microfinance schemes, mostly group savings and loans approaches, which could be complemented by other intervention components, including gender dialog groups (one study), multiple components for couples, communities, supportive environments, and victim support (one study). Community mobilization encompassed interventions that trained community activists and community leaders to engage communities in critical reflection (one study), trained outreach workers to facilitate reflection sessions, trained male champions and built alliances (one study), or encompassed multiple components to intervene at the individual, relationship, and societal levels (one study). IPV screening and referral were undertaken in a single session by trained health care providers and could include safety planning, harm reduction counseling and a booster session (one study), an information card (one study), or improvement of service delivery, strengthening of linkages between services, community sensitization, group education, and couples’ skills building (one study).

A minority of studies included a no-intervention control group. Most studies included a usual care or basic intervention comparison group. Few studies included an attention control condition. The purpose of most interventions was to contribute to the prevention of violence against women. Other interventions were conducted with the purpose of HIV prevention (seven studies), promoting maternal and reproductive health or child health (three studies), or preventing hazardous alcohol use (one study). Just over half of the studies tested an intervention that was explicitly noted to be informed by theory and/or by conceptually informed change approaches. Studies assessed various types of IPV, including any IPV, physical IPV, sexual IPV, physical and/or sexual IPV, and emotional IPV. Most (10) studies reported on one IPV outcome, two reported on two outcomes, five each on three and four outcomes, one on five outcomes, two on six outcomes, and one on eight outcomes. While nearly all studies (also) included outcomes reflecting IPV experienced by women, fewer (also) assessed the perpetration of IPV as reported by men. Most studies assessed the occurrence of IPV over a period of 6–12 months post-intervention and included a (last) follow-up more than 1 year after completion of the intervention. Participant assessments were interviewer assisted in most of the included studies. The WHO Violence Against Women scale (García-Moreno et al., 2005) was most often used to assess IPV, followed by the revised Conflict Tactics Scale (CTS2; Straus et al., 1996). Two studies did not mention the use of a preexisting instrument to assess IPV. All studies reported intent-to-treat analyses and all but one study explicitly noted that analyses were adjusted for baseline scores on outcome measures. Analyses were generally also adjusted for potential confounders, including (baseline) sample differences.

### Intervention Types by Country and Target Groups

Table 2 shows the crosstabulation of IPV prevention interventions by the country setting in which these were tested (low- and middle-income countries vs. high-income countries) and the population group of the intervention (women only vs. women and men or men only). This explorative analysis showed that the effects of individual support/counseling interventions were mostly tested in high-income countries. In contrast, the effects of interventions consisting of small-group counseling, economic empowerment, or community mobilization were predominantly or exclusively tested in low- and middle-income countries. Effects of IPV screening and referral interventions were mostly tested in low- and middle-income countries. Furthermore, we found that the effects of individual support/counseling interventions were almost exclusively tested in women. Effects of small-group counseling, economic empowerment, and community mobilization interventions were we predominantly or almost exclusively tested in women. Research on the effects of IPV screening and referral interventions did not involve men.

**Table 2.** IPV Prevention Intervention Effect Sizes Tested by Country Setting and Target Group.

Intervention type	Country setting		Target group	
	LMIC	HIC	Women only	Women and men or men only
Individual support/counseling	*n* = 3	*n* = 6	*n* = 8	*n* = 1
Small-group counseling	*n* = 42	*n* = 4	*n* = 14	*n* = 32
Economic empowerment	*n* = 15	*n* = 0	*n* = 5	*n* = 10
Community mobilization	*n* = 11	*n* = 0	*n* = 1	*n* = 10
IPV screening and referral	*n* = 7	*n* = 3	*n* = 10	*n* = 0

*Note.* IPV = intimate partner violence.

### Risk of Bias

**Figure 2.**
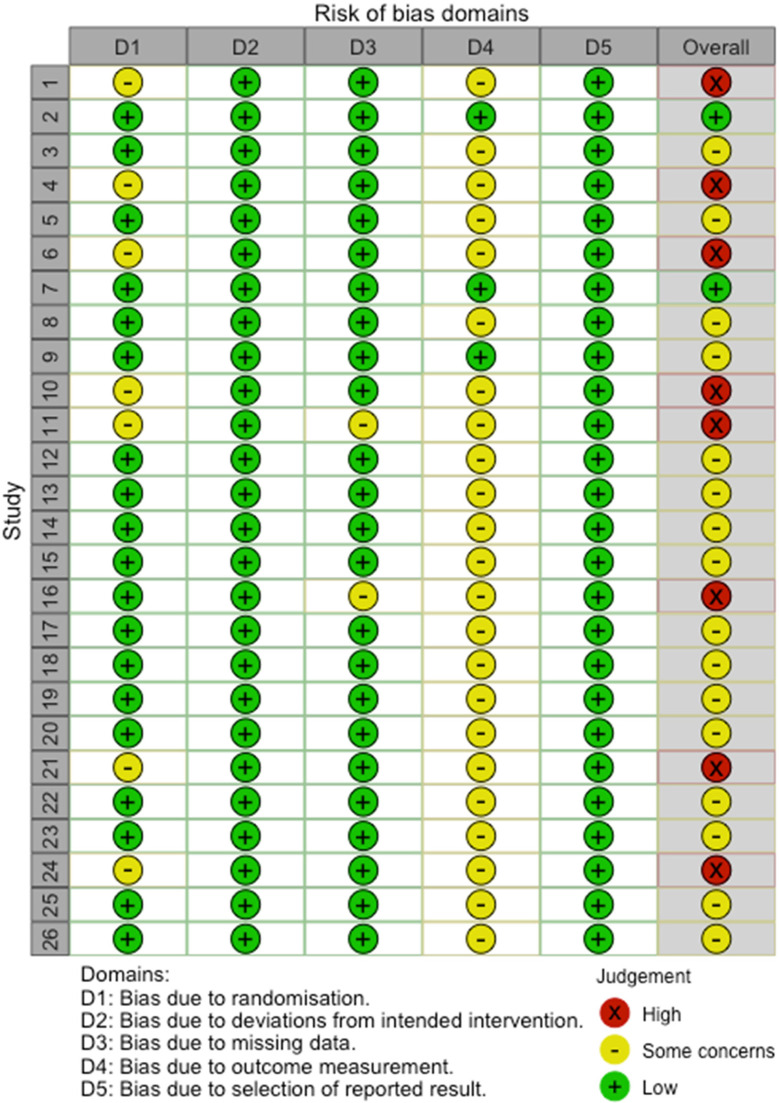
Risk of Bias Assessment of Individual Studies.

Note: study numbers refer to included articles: 1 = Abramsky et al. (2016); 2 = Decker et al. (2020); 3 = Doyle et al. (2018); 4 = Dunkle et al. (2020); 5 = Gibbs et al. (2020); 6 = Gibbs, Washington et al. (2020); 7 = Gilbert et al. (2016); 8 = Gupta et al. (2013); 9 = Gupta et al. (2017); 10 = Harvey et al. (2021); 11 = Javalkar et al. (2019); 12 = Kapiga et al. (2019); 13 = Kielyet al. (2010); 14 = Koziol-McLain et al. (2010); 15 = MacMillan et al. (2009); 16 = Maman et al. (2020); 17 = Miller et al. (2016); 18 = Murray et al. (2020); 19 = Rhodes et al. (2015); 20 = Salazar et al. (2014); 21 = Settergren et al. (2018); 22 = Sharma et al. (2020); 23 = Taft et al. (2011); 24 = Vaillant et al. (2020); 25 = Van Parys et al. (2017); 26 = Wagman et al. (2015).

As shown in Figure 2, most studies scored low on all RoB dimensions. Nevertheless, only two studies received an overall low RoB rating. This reflects that most studies received a rating of some concerns on one or more dimensions, resulting in an overall RoB scores of some concerns or high concern. For all but three studies, RoB related to the outcome assessment was rated as entailing some concerns, due to the nonblinding of participants to study condition. The seven studies rated as entailing some concerns with respect to RoB related to randomization were all cRCTs that did not recruit/identify all participants before randomization of the clusters. The two studies rated as entailing some concerns with respect to RoB related to missing data both reported differential missingness that could be related to the study outcome(s). We found that all studies were at low RoB with respect to deviations from the intended interventions, which we assessed with respect to assignment to intervention, as this aligns with the intention-to-treat approach to data analysis reported by all studies.

### Evidence Synthesis

Table 3 presents an overview of included effects. As can be seen, reported effects differed substantially. We found 22 significant effects across 12 studies. Using multilevel meta-analysis, we found a significant and beneficial pooled intervention effect: RR = 0.85, 95% CI [0.77, 0.99]. Intervention effects were heterogeneous (Total *I*^2^ = 83.6%). Of the total variance, 16.4% was estimated to be related to sampling error. Within-study study variance (i.e., variance due to differences in IPV outcomes) was estimated to be limited (3.4%). Most of the estimated variance in effects (80.2%) was attributed to between-study differences. A comparison of the simple and multilevel random effects models to estimate the pooled intervention effects showed that the multilevel model had a better fit (χ^2^ = 53.6, *p* < .001).

**Table 3.** Overview of effect sizes

*Study - outcome (intervention arm)*	Relative Risk	*95% Confidence Interval*	
		Lower bound	Upper bound
Abramsky et al. (2016) - Experienced Physical IPV	0.48	0.16	1.39
Abramsky et al. (2016) - Experienced Sexual IPV	0.76	0.33	1.72
Abramsky et al. (2016) - Experienced Physical and/or Sexual IPV	0.69	0.35	1.38
Abramsky et al. (2016) - Experienced Emotional IPV	0.61	0.47	0.79
Decker et al. (2020) - Experienced Physical IPV	0.57	0.09	3.08
Decker et al. (2020) - Experienced Sexual IPV	1.33	0.69	2.23
Decker et al. (2020) - Experienced Physical & Sexual IPV	1.06	0.88	1.18
Doyle et al. (2018) - Experienced Physical IPV	0.57	0.47	0.69
Doyle et al. (2018) - Experienced Sexual IPV	0.56	0.46	0.70
Dunkle et al. (2020) - Experienced Physical IPV	0.39	0.29	0.54
Dunkle et al. (2020) - Experienced Sexual IPV	0.49	0.37	0.66
Dunkle et al. (2020) - Experienced Physical and/or Sexual IPV	0.44	0.34	0.59
Dunkle et al. (2020) - Perpetrated Physical IPV	0.78	0.56	1.09
Dunkle et al. (2020) - Perpetrated Sexual IPV	0.52	0.37	0.74
Dunkle et al. (2020) - Perpetrated Physical and/or Sexual IPV	0.54	0.38	0.75
Gibbs et al. (2020) - Experienced Physical IPV	0.91	0.69	1.16
Gibbs. Washington et al. (2020) - Experienced Physical IPV	0.96	0.78	1.14
Gibbs. Washington et al. (2020) - Experienced Sexual IPV	0.93	0.73	1.17
Gibbs. Washington et al. (2020) - Perpetrated Physical IPV	0.81	0.65	0.98
Gibbs. Washington et al. (2020) - Perpetrated Sexual IPV	0.80	0.62	1.02
Gilbert et al. (2016) - Experienced Physical IPV (computerized materials arm)	0.43	0.18	0.97
Gilbert et al. (2016) - Experienced Sexual IPV (computerized materials arm)	0.58	0.20	1.55
Gilbert et al. (2016) - Experienced Physical IPV (no computerized materials arm)	0.71	0.35	1.34
Gilbert et al. (2016) - Experienced Sexual IPV (no computerized materials arm)	0.88	0.39	1.87
Gupta et al. (2013) - Experienced Physical IPV	0.72	0.43	1.17
Gupta et al. (2013) - Experienced Sexual IPV	0.74	0.44	1.21
Gupta et al. (2013) - Experienced Physical and/or Sexual IPV	1.14	0.74	1.68
Gupta et al. (2017) - Experienced Physical IPV	1.15	0.81	1.40
Gupta et al. (2017) - Experienced Sexual IPV	0.92	0.63	1.29
Gupta et al. (2017) - Experienced Physical and/or Sexual IPV	0.82	0.55	1.18
Harvey et al. (2021) - Experienced Physical IPV	0.98	0.76	1.25
Harvey et al. (2021) - Experienced Sexual IPV	0.98	0.77	1.24
Harvey et al. (2021) - Experienced Emotional IPV	0.84	0.70	0.99
Javalkar et al. (2019) - Experienced Physical and/or Sexual IPV	1.50	0.73	2.59
Kapiga et al. (2019) - Experienced Physical and/or Sexual IPV	0.75	0.55	1.01
Kapiga et al. (2019) - Experienced Physical IPV	0.69	0.46	0.99
Kapiga et al. (2019) - Experienced Sexual IPV	0.83	0.56	1.20
Kapiga et al. (2019) - Experienced Emotional IPV	0.99	0.81	1.19
Kiely et al. (2010) - Experienced Sexual IPV	0.99	0.50	1.84
Kiely et al. (2010) - Experienced Physical IPV	0.55	0.34	0.86
Koziol-McLain et al. (2010) - Any Experienced IPV	0.88	0.43	1.70
MacMillan et al. (2009) - Any Experienced IPV	0.91	0.50	1.33
Maman et al. (2020) - Perpetrated Physical and/or Sexual IPV	1.14	0.91	1.44
Miller et al. (2016) - Experienced Physical and/or Sexual IPV	1.07	0.84	1.38
Murray et al. (2020) - Experienced Physical IPV	0.75	0.57	0.99
Murray et al. (2020) - Experienced Sexual IPV	0.56	0.48	0.88
Murray et al. (2020) - Perpetrated Physical IPV	0.73	0.54	0.98
Murray et al. (2020) - Perpetrated Sexual IPV	0.68	0.46	1.00
Rhodes et al. (2015) - Any Experienced IPV	1.01	0.99	1.04
Salazar et al. (2014) - Any Perpetrated IPV	0.35	0.15	0.77
Settergren et al. (2018) - Any Experienced IPV	0.91	0.75	1.08
Settergren et al. (2018) - Experienced Physical IPV	0.99	0.76	1.24
Settergren et al. (2018) - Experienced Sexual IPV	0.77	0.56	1.04
Settergren et al. (2018) - Experienced Emotional IPV	0.86	0.68	1.06
Sharma et al. (2020) - Experienced Physical IPV (women's arm)	1.09	0.89	1.31
Sharma et al. (2020) - Experienced Sexual IPV (women's arm)	1.09	0.93	1.27
Sharma et al. (2020) - Experienced Physical and/or Sexual IPV (women's arm)	1.05	0.90	1.20
Sharma et al. (2020) - Experienced Emotional IPV (women's arm)	0.96	0.83	1.10
Sharma et al. (2020) - Perpetrated Physical IPV (women's arm)	1.16	0.90	1.46
Sharma et al. (2020) - Perpetrated Sexual IPV (women's arm)	1.05	0.88	1.24
Sharma et al. (2020) - Perpetrated Physical and/or Sexual IPV (women's arm)	1.10	0.94	1.26
Sharma et al. (2020) - Perpetrated Emotional IPV (women's arm)	1.08	0.97	1.19
Sharma et al. (2020) - Experienced Physical IPV (men's arm)	1.02	0.84	1.21
Sharma et al. (2020) - Experienced Sexual IPV (men's arm)	0.86	0.73	1.01
Sharma et al. (2020) - Experienced Physical and/or Sexual IPV (men's arm)	0.88	0.77	0.99
Sharma et al. (2020) - Experienced Emotional IPV (men's arm)	0.92	0.81	1.02
Sharma et al. (2020) - Perpetrated Physical IPV (men's arm)	0.88	0.70	1.07
Sharma et al. (2020) - Perpetrated Sexual IPV (men's arm)	0.79	0.64	0.96
Sharma et al. (2020) - Perpetrated Physical and/or Sexual IPV (men's arm)	0.85	0.73	0.99
Sharma et al. (2020) - Perpetrated Emotional IPV (men's arm)	0.99	0.89	1.09
Sharma et al. (2020) - Experienced Physical IPV (couples' arm)	1.00	0.81	1.23
Sharma et al. (2020) - Experienced Sexual IPV (couples' arm)	0.91	0.72	1.12
Sharma et al. (2020) - Experienced Physical and/or Sexual IPV (couples' arm)	0.92	0.77	1.08
Sharma et al. (2020) - Experienced Emotional IPV (couples' arm)	0.97	0.83	1.10
Sharma et al. (2020) - Perpetrated Physical IPV (couples' arm)	0.98	0.75	1.25
Sharma et al. (2020) - Perpetrated Sexual IPV (couples' arm)	0.90	0.69	1.17
Sharma et al. (2020) - Perpetrated Physical and/or Sexual IPV (couples' arm)	0.92	0.73	1.12
Sharma et al. (2020) - Perpetrated Emotional IPV (couples' arm)	1.00	0.88	1.11
Taft et al. (2011) - Any Experienced IPV	0.71	0.43	1.02
Vaillant et al. (2020) - Experienced Physical IPV	0.92	0.74	1.07
Vaillant et al. (2020) - Experienced Sexual IPV	0.99	0.78	1.16
Vaillant et al. (2020) - Experienced Physical and/or IPV	0.97	0.81	1.10
Vaillant et al. (2020) - Experienced Emotional IPV	0.97	0.86	1.05
Vaillant et al. (2020) - Any Experienced IPV	0.99	0.89	1.06
Van Parys et al. (2017) - Any Experienced IPV	1.09	0.66	1.62
Wagman et al. (2015) - Experienced Physical IPV	0.79	0.67	0.92
Wagman et al. (2015) - Experienced Sexual IPV	0.80	0.67	0.97
Wagman et al. (2015) - Experienced Emotional IPV	0.91	0.79	1.04
Wagman et al. (2015) - Perpetrated Physical IPV	1.00	0.77	1.30
Wagman et al. (2015) - Perpetrated Sexual IPV	0.81	0.52	1.26
Wagman et al. (2015) - Perpetrated Emotional IPV	0.99	0.85	1.16

We undertook a series of univariate meta-regression analyses to assess main study characteristics associated with effect sizes. As shown in Table 4, only the gender of intervention participants was significantly associated with intervention effects. Interventions that included women and men or men only were significantly more effective than interventions that only included women.

**Table 4.** Univariate Metaregression Analyses of IPV Prevention Intervention Effects on Study Characteristics.

Study characteristic	*n* of effects	*Z* coefficient	*p*
Publication year ..2008–2016 2017–2022	24 67	Reference [REF]0.13	.21
Start year ..2001–2013 2014–2018	33 58	Reference 0.00	.99
Country High income Low/middle income	13 78	Reference 0.01	.95
Location Urban Rural	40 51	Reference −0.07	.49
Setting Health care Community	16 75	Reference −0.14	.19
Design ..RCT CRCT	24 67	Reference 0.08	.41
Population Women only ..Women and men/men only	38 53	Reference −0.14	.0001
Experience or risk of IPV No ..Yes	76 15	Reference 0.03	.82
Participants .. < 1,000 .. > 1,000	24 67	Reference −0.07	.49
Intervention ..Individual support/counseling ..Small-group counseling ..Economic empowerment ..Community mobilization ..IPV screening and referral	9 46 15 11 10	Reference −0.10 −0.07 −0.04 0.08	.83
Control condition ..No intervention/waitlist ..Usual care/basic intervention ..Attention control intervention	14 67 10	Reference −0.30 −0.60	.57
Purpose ..IPV prevention ..Other main purpose	35 56	Reference −0.08	.17
Theory informed ..No ..Yes	51 40	Reference −0.11	.27
IPV outcome ..Any IPV ..Physical IPV ..Sexual IPV ..Physical and/or sexual IPV ..Emotional IPV	8 27 26 17 13	Reference −0.02 −0.08 −0.05 −0.01	.55
Actor perspective ..Experienced IPV ..Perpetrated IPV	67 24	Reference 0.04	.20
Assessment period ..≤ 6 months ..6–12 months	14 77	Reference −0.16	.13
Follow-up period .. ≤12 months .. > 12 months	23 68	Reference −0.07	.49
Data collection ..Self-administered ..Interviewer assisted	27 64	Reference −0.12	.24
IPV measure ..VAW scale ..Other/not-identified measure	61 30	Reference 0.04	.71
Risk of bias (overall) Low/some concerns ..High	63 28	Reference −0.01	.90

*Note.* IPV = intimate partner violence.

## Discussion

Twenty-six studies, contributing 91 effects of interventions to prevent the occurrence of experienced or perpetrated IPV, were included in this meta-analysis. Most studies were conducted in urban settings and in low- and middle-income countries, in particular sub-Saharan Africa. Study design, setting, participants, intervention types, control or comparison conditions, outcomes and measures, assessment periods and follow-up durations differed substantially between studies. Also, the overall RoB was substantial across the included studies, with only two studies receiving a low RoB score. Some concerns regarding RoB were mostly related to the use of self-report measures to assess outcomes among participants who were not blinded to the study condition they were randomized to, which was related to the nature of the study design, including the requirement for community approvals (e.g., Dunkle et al., 2020), and/or the type of interventions tested (e.g., Doyle et al., 2018; Van Parys et al., 2017), to the content of which participants would be inherently aware. This also precluded blinding intervention deliverers to study conditions (e.g., Javalkar et al., 2019; also see Kapiga et al., 2019; Maman et al., 2020; Murray et al., 2020). Data were frequently collected by interviewers who were also not blinded to study conditions (e.g., Abramsky et al., 2016; Sharma et al., 2020). Data analysts also were not always blinded (e.g., Gibbs et al., 2020). Most designs of cluster randomized controlled trails additionally raised some concerns regarding RoB related to the identification or selection of participants after the randomization of clusters, which generally seemed more practical. It was also noted that postrandomization recruitment was undertaken to mitigate “mistrust among marginalized communities around research, and historical experiences of communities being promised support and then not receiving it,” (Gibbs et al., 2020, p. 326).

Significant effects were reported in almost half of the studies, albeit effects were significant for less than a quarter of the assessed outcomes. We nevertheless found a significant pooled effect of RR = 0.85, 95% CI [0.77, 0.99], corresponding to an average reduction of 15% in intervention participants’ risk of experiencing or perpetrating IPV. This overall significant effect is aligned with the observation that evidence supports the efficacy of various approaches to prevent the occurrence of IPV (Jewkes et al., 2021), and provides a quantitative indication of the size of this effect. The statistical significance of the pooled effect does, however, not signify that any intervention will be effective in each country setting and for each target group. As we showed, the types of interventions tested differ between low- and middle-income countries and high-income countries, and interventions tested also differed for women only or for women and men or men only. These differences may reflect that interventions are developed and implemented based on a theory of change, which takes the characteristics of the local contexts into account (see Jewkes et al., 2021), as well as that of the population group. While just over half of the studies included in the meta-analyses reported that interventions were guided by theory, little details were provided about these theories and, more importantly, how they guided the intervention approach. Typically, little information was provided about any theory of change that may have guided the intervention. As Jewkes et al. (2021, p. 2) note, “descriptions of the design and implementation of interventions are often thin.” It is also possible intervention developers, policymakers, sponsors, and/or beneficiary communities have preferred practices and established expectations that may limit the types of interventions considered for development or implementation, suggesting there may be scope for learning by adapting and comparing interventions, including their feasibility, acceptability, and effects, across country settings and population groups.

We found that intervention effects were significantly heterogeneous, highlighting the importance of assessing the role of factors that may be associated with effects. Of the wide range of study characteristics assessed as potential effect modifiers in metaregression analysis, we only found that the gender of intervention participants was related to a difference in intervention effects, such that interventions for women and men or for men only were more effective than interventions for women only. There is a noted increase in interest in IPV prevention interventions for men (Graham et al., 2021), and interventions (also) targeting (potential) perpetrators may be more effective than interventions that only target (potential) victims. However, we also found that the type of interventions tested among women and men or men only differed from those tested among women only. Interventions for women only mostly consisted of individual support/counseling and IPV screening in health care settings, which may be less aligned with the main drivers of IPV than interventions (also) including men that tended to consist of community mobilization and small group counseling. The theory of change of interventions for women only may hence be less appropriate (cf. Jewkes et al., 2021).

We did not find a significant difference between the effects of interventions tested in low- and middle-income countries or in high-income countries. This illustrates that effective interventions are now available for low- and middle-income country settings, where the prevalence of IPV tends to be higher (cf. Sardinha et al., 2022). A systematic review of IPV prevention interventions in sub-Saharan Africa equally found evidence of beneficial effects (Cork et al., 2020), albeit that they included few effects, and the size of the effect and their association with potential effect modifiers was not quantified. We also did not observe significant differences according to the type of intervention, but, as already noted, these differed substantially between country settings and target groups and more research is hence needed to build a more robust evidence base to guide the selection of the most promising intervention type in various country settings and for different population groups. It is also of note that we did not find an association between intervention effects and the type of outcome assessed. This suggests that IPV prevention interventions have the potential to affect a range of outcomes, that is, physical, sexual, and emotional IPV, which are likley closely aligned. Our analyses of different sources of variance in intervention effects similarly found that differences between IPV outcomes assessed did not account for much difference in intervention effects observed. This underscores that the inclusion of multiple outcomes in analyses of intervention effects contains little risk of amounting to fishing for effects, as effects across outcomes are similar. Lastly, RoB was also not associated with the effect of interventions. This may be due to the limited difference in RoB between studies.

A number of strengths and limitations should be considered with respect to the interpretation of the results of this study. Strengths include that our evidence synthesis builds on a review of research conducted untill 2008 (World Health Organization/London School of Hygiene and Tropical Medicine, 2010). Also, we extended existing systematic reviews of IPV intervention studies by applying a broad rather than specific focus and compared the effects of different types of interventions, for different population groups and in different regions. Furthermore, we extended current evidence syntheses by undertaking a meta-analysis, considered the highest level of evidence to guide health care decisions (Guyatt et al., 1995), which provided a quantitative estimate of the effect of preventive interventions on the occurrence of any experience or perpetration of IPV. We also uniquely used a multilevel meta-analysis approach to synthesize findings of studies testing the effects of IP prevention interventions. This underutilized approach enabled us to make maximum use of information on effects and robustly assess potential effect modifiers. Limitations include that, while our evidence synthesis had a broad scope, including in terms of country settings, population groups and intervention types, we did not include studies published before 2008 and only included published research indexed in four major scholarly literature databases. This may have resulted in some publications missed, albeit we undertook an extensive search of references in and to included papers and systematic reviews. Also, we applied specific in- and exclusion criteria and only focused on studies with outcomes indicating any experience or perpetration of IPV. We excluded studies reporting the frequency and/or severity of IPV outcomes that could not be transformed into indicators of any occurrence of IPV. In addition, we only included studies for which sufficient details on sample size calculation were reported, ensuring that included studies were fit for the purpose of testing the effects of IPV intervention on the occurrence of IPV.

IPV remains a widespread social problem, with far-reaching repercussions for women's health and well-being globally. The findings of this meta-analysis underscore that the risk of IPV can be effectively mitigated through preventive interventions, which should be tailored to the requirements and constraints of specific population groups and contexts to maximize impact and allow scaling up (see Jewkes et al., 2021). Given the current historical moment, with continued attention on IPV and broader gender-based violence and discrimination due to the #metoo movements, researchers, program developers, and policymakers, have a unique opportunity and responsibility to make the most of this attention to further build evidence and, in particular, implement and bring to scale proven approaches to address the pervasive problem of IPV. This is critical to accelerate progress toward sexual and reproductive health and rights for all, as called for by the Guttmacher–Lancet Commission (Starrs et al., 2018), and to attain Sustainable Development Goal 5.2, to eliminate all forms of violence against all women and girls in the public and private spheres, including trafficking and sexual and other types of exploitation.

## References

[bibr1-10778012231183660] Abramsky T. Devries K. M. Michau L. Nakuti J. Musuya T. Kyegombe N. Watts C. (2016). The impact of SASA!, a community mobilisation intervention, on women’s experiences of intimate partner violence: Secondary findings from a cluster randomised trial in Kampala, Uganda. Journal of Epidemiology and Community Health, 70(8), 818–825. 10.1136/jech-2015-206665 26873948 PMC4975800

[bibr2-10778012231183660] Abramsky T. Watts C. H. Garcia-Moreno C. Devries K. Kiss L. Ellsberg M. Jansen H. A. F. M. Heise L. (2011). What factors are associated with recent intimate partner violence? Findings from the WHO multi-country study on women’s health and domestic violence. BMC Public Health, 11, 109. 10.1186/1471-2458-11-109 21324186 PMC3049145

[bibr3-10778012231183660] Anderson J. C. Campbell J. C. Farley J. E. (2013). Interventions to address HIV and intimate partner violence in sub-Saharan Africa: A review of the literature. Journal of the Association of Nurses in AIDS Care, 24(4), 383–390. 10.1016/j.jana.2013.03.003 PMC3694280 23790280

[bibr4-10778012231183660] Assink M. Wibbelink C. J. M. (2016). Fitting three-level meta-analytic models in R: A step-by-step tutorial. Quantitative Methods for Psychology, 12(3), 154–174. https://doi.org/ 10.20982/tqmp.12.3.p154

[bibr5-10778012231183660] Campbell J. C. (2002). Health consequences of intimate partner violence. Lancet, 359(9314), 1331–1336. 10.1016/S0140-6736(02)08336-8 11965295

[bibr6-10778012231183660] Cheung M. W.-L. (2014). Modeling dependent effect sizes with three-level meta-analyses: A structural equation modeling approach. Psychological Methods, 19(2), 211–229. 10.1037/a0032968 23834422

[bibr7-10778012231183660] Cheung M. W.-L. (2019). A guide to conducting a meta-analysis with non-independent effect sizes. Neuropsychology Review, 29(4), 387–396. 10.1007/s11065-019-09415-6 31446547 PMC6892772

[bibr8-10778012231183660] Cork C. White R. Noel P. Bergin N. (2020). Randomized controlled trials of interventions addressing intimate partner violence in sub-Saharan Africa: A systematic review. Trauma, Violence and Abuse, 21(4), 643–659. 10.1177/1524838018784585 PMC7197024 29962286

[bibr9-10778012231183660] Decker M. R. Wood S. N. Hameeduddin Z. Kennedy S. R. Perrin N. Tallam C. Akumu I. Wanjiru I. Asira B. Frankel A. Omondi B. Case J. Clough A. Otieno R. Mwiti M. Glass N. (2020). Safety decision-making and planning mobile app for intimate partner violence prevention and response: Randomised controlled trial in Kenya. BMJ Global Health, 5(7), Article e002091. 10.1136/bmjgh-2019-002091 PMC7368487 32675229

[bibr10-10778012231183660] Doyle K. Levtov R. G. Barker G. Bastian G. G. Bingenheimer J. B. Kazimbaya S. Nzabonimpa A. Pulerwitz J. Sayinzoga F. Sharma V. Shattuck D. (2018). Gender-transformative Bandebereho couples’ intervention to promote male engagement in reproductive and maternal health and violence prevention in Rwanda: Findings from a randomized controlled trial. PloS One, 13(4), Article e0192756. 10.1371/journal.pone.0192756 PMC5884496 29617375

[bibr11-10778012231183660] Dunkle K. Stern E. Chatterji S. Heise L. (2020). Effective prevention of intimate partner violence through couples training: A randomised controlled trial of Indashyikirwa in Rwanda. BMJ Global Health, 5(12), Article e002439. 10.1136/bmjgh-2020-002439 PMC7757483 33355268

[bibr12-10778012231183660] Dunkle K. L. Decker M. R. (2013). Gender-based violence and HIV: Reviewing the evidence for links and causal pathways in the general population and high-risk groups. American Journal of Reproductive Immunology, 69(Suppl 1), 20–26. 10.1111/aji.12039 23216606

[bibr13-10778012231183660] Eggers Del Campo I. Steinert J. I. (2022). The effect of female economic empowerment interventions on the risk of intimate partner violence: A systematic review and meta-analysis. Trauma, Violence and Abuse, 23(3), 810–826. 10.1177/1524838020976088 33287669

[bibr14-10778012231183660] Ellsberg M. Jansen H. A. F. M. Heise L. Watts C. H. Garcia-Moreno C. ; WHO Multi-country Study on Women’s Health and Domestic Violence against Women Study Team (2008). Intimate partner violence and women’s physical and mental health in the WHO multi-country study on women’s health and domestic violence: An observational study. Lancet, 371(9619), 1165–1172. 10.1016/s0140-6736(08)60522-x 18395577

[bibr15-10778012231183660] García-Moreno C. Jansen H. A. F. M. Ellsberg M. Heise L. Watts C. (2005). WHO multi-country study on women’s health and domestic violence against women: Initial results on prevalence, health outcomes and women’s responses. World Health Organization.

[bibr16-10778012231183660] García-Moreno C. Zimmerman C. Morris-Gehring A. Heise L. Amin A. Abrahams N. Montoya O. Bhate-Deosthali P. Kilonzo N. Watts C. (2015). Addressing violence against women: A call to action. Lancet, 385(9978), 1685–1695. 10.1016/S0140-6736(14)61830-4 25467579

[bibr17-10778012231183660] Gibbs A. Corboz J. Chirwa E. Mann C. Karim F. Shafiq M. Mecagni A. Maxwell-Jones C. Noble E. Jewkes R. (2020). The impacts of combined social and economic empowerment training on intimate partner violence, depression, gender norms and livelihoods among women: An individually randomised controlled trial and qualitative study in Afghanistan. BMJ Global Health, 5(3), Article e001946. 10.1136/bmjgh-2019-001946 PMC7076232 32201622

[bibr18-10778012231183660] Gibbs A. Washington L. Abdelatif N. Chirwa E. Willan S. Shai N. Sikweyiya Y. Mkhwanazi S. Ntini N. Jewkes R. (2020). Stepping stones and creating futures intervention to prevent intimate partner violence among young people: Cluster randomized controlled trial. Journal of Adolescent Health, 66(3), 323–335. 10.1016/j.jadohealth.2019.10.004 31784410

[bibr19-10778012231183660] Gilbert L. Goddard-Eckrich D. Hunt T. Ma X. Chang M. Rowe J. McCrimmon T. Johnson K. Goodwin S. Almonte M. Shaw S. A. (2016). Efficacy of a computerized intervention on HIV and intimate partner violence among substance-using women in community corrections: A randomized controlled trial. American Journal of Public Health, 106(7), 1278–1286. 10.2105/AJPH.2016.303119 27077342 PMC4984779

[bibr20-10778012231183660] Graham L. M. Embry V. Young B.-R. Macy R. J. Moracco K. E. McNaughton Reyes H. L. Martin S. L. (2021). Evaluations of prevention programs for sexual, dating, and intimate partner violence for boys and men: A systematic review. Trauma, Violence and Abuse, 22(3), 439–465. 10.1177/1524838019851158 31262233

[bibr21-10778012231183660] Gupta J. Falb K. L. Lehmann H. Kpebo D. Xuan Z. Hossain M. Zimmerman C. Watts C. Annan J. (2013). Gender norms and economic empowerment intervention to reduce intimate partner violence against women in rural Côte d’Ivoire: A randomized controlled pilot study. BMC International Health and Human Rights, 13, Article 46. 10.1186/1472-698X-13-46 24176132 PMC3816202

[bibr22-10778012231183660] Gupta J. Falb K. L. Ponta O. Xuan Z. Abril Campos P. Arellano Gomez A. Valades J. Cariño G. Diaz Olavarrieta C. (2017). A nurse-delivered, clinic-based intervention to address intimate partner violence among low-income women in Mexico City: Findings from a cluster randomized controlled trial. BMC Medicine, 15(1), Article 128. 10.1186/s12916-017-0880-y 28697769 PMC5506677

[bibr23-10778012231183660] Guyatt G. H. Sackett D. L. Sinclair J. C. Hayward R. Cook D. J. Cook R. J. (1995). Users’ guides to the medical literature. IX. A method for grading health care recommendations. Journal of the American Medical Association, 274(22), 1800–1804. 10.1001/jama.274.22.1800 7500513

[bibr24-10778012231183660] Harrer M. Cuijpers P. Furukawa T. Ebert D. D. (2019). *Dmetar: companion R package for the guide ‘doing meta-analysis in R’* (R package Version 0.0.9000). http://dmetar.protectlab.org/.

[bibr25-10778012231183660] Harrer M. Cuijpers P. Furukawa T. A. Ebert D. D. (2021). Doing meta-analysis with R: A hands-on guide. Chapman & Hall/CRC Press. 10.1201/9781003107347

[bibr26-10778012231183660] Harvey S. Abramsky T. Mshana G. Holm Hansen C. Mtolela G. J. Madaha F. Hashim R. Kapinga I. Watts C. Lees S. Kapiga S. (2021). A cluster randomized controlled trial to evaluate the impact of a gender transformative intervention on intimate partner violence against women in newly formed neighbourhood groups in Tanzania. BMJ Global Health, 6(7), Article e004555. 10.1136/bmjgh-2020-004555 PMC8311325 34301673

[bibr27-10778012231183660] Hedges L. V. Vevea J. L. (1998). Fixed- and random-effects models in meta-analysis. Psychological Methods, 3(4), 486–504. 10.1037/1082-989X.3.4.486

[bibr28-10778012231183660] Heise L. (2011). What works to prevent partner violence? An evidence overview. London School of Hygiene and Tropical Medicine.

[bibr29-10778012231183660] Hines D. A. (2007). Predictors of sexual coercion against women and men: A multilevel, multinational study of university students. Archives of Sexual Behavior, 36(3), 403–422. 10.1007/s10508-006-9141-4 17333324

[bibr30-10778012231183660] Janssen P. A. Holt V. L. Sugg N. K. Emanuel I. Critchlow C. M. Henderson A. D. (2003). Intimate partner violence and adverse pregnancy outcomes: A population-based study. American Journal of Obstetrics and Gynecology, 188(5), 1341–1347. 10.1067/mob.2003.274 12748509

[bibr31-10778012231183660] Javalkar P. Platt L. Prakash R. Beattie T. S. Collumbien M. Gafos M. Ramanaik S. Davey C. Jewkes R. Watts C. Bhattacharjee P. Thalinja R. Kavitha D. L. Isac S. Heise L. (2019). Effectiveness of a multilevel intervention to reduce violence and increase condom use in intimate partnerships among female sex workers: Cluster randomised controlled trial in Karnataka, India. BMJ Global Health, 4(6), Article e001546. 10.1136/bmjgh-2019-001546 PMC6861099 31798984

[bibr32-10778012231183660] Jewkes R. (2002). Intimate partner violence: Causes and prevention. Lancet, 359(9315), 1423–1429. 10.1016/S0140-6736(02)08357-5 11978358

[bibr33-10778012231183660] Jewkes R. Willan W. Heise L. Washington L. Shai N. Kerr-Wilson A. Gibbs A. Stern E. Christofides N. (2021). Elements of the design and implementation of interventions to prevent violence against women and girls associated with success: Reflections from the what works to prevent violence against women and girls? Global programme. International Journal of Environmental Research and Public Health, 18(22), Article 12129. 10.3390/ijerph182212129 34831885 PMC8621962

[bibr34-10778012231183660] Kapiga S. Harvey S. Mshana G. Holm Hansen C. Mtolela G. J. Madaha F. Hashim R. Kapinga I. Mosha N. Abramsky T. Lees S. Watts C. (2019). A social empowerment intervention to prevent intimate partner violence against women in a microfinance scheme in Tanzania: Findings from the MAISHA cluster randomised controlled trial. Lancet Global Health, 7(10), e1423–e1434. 10.1016/S2214-109X(19)30316-X 31537372

[bibr35-10778012231183660] Kiely M. El-Mohandes A. A. E. El-Khorazaty M. N. Gantz M. G. (2011). An integrated intervention to reduce intimate partner violence in pregnancy: A randomized trial. Obstetrics and Gynecology, 115(2 pt 1), 273–283. 10.1097/AOG.0b013e3181cbd482 PMC2917915 20093899

[bibr36-10778012231183660] Kouyoumdjian F. G. Findlay N. Schwandt M. Calzavara L. M. (2013). A systematic review of the relationships between intimate partner violence and HIV/AIDS. PloS One, 8, 1–25. 10.1371/journal.pone.0081044 PMC3840028 24282566

[bibr37-10778012231183660] Koziol-Mclain J. Garrett N. Fanslow J. Hassall I. Dobbs T. Henare-Toka T. A. Lovell V. (2010). A randomized controlled trial of a brief emergency department intimate partner violence screening intervention. Annals of Emergency Medicine, 56(4), 413–423.e1. 10.1016/j.annemergmed.2010.05.001 20538369

[bibr38-10778012231183660] MacMillan H. L. Wathen C. N. Jamieson E. Boyle M. H. Shannon H. S. Ford-Gilboe M. Worster A. Lent B. Coben J. H. Campbell J. C. McNutt L.-A. ; McMaster Violence Against Women Research Group. (2009). Screening for intimate partner violence in health care settings: A randomized trial. Journal of the American Medical Association, 302(5), 493–501. 10.1001/jama.2009.1089 19654384

[bibr39-10778012231183660] Maman S. Mulawa M. I. Balvanz P. McNaughton Reyes H. L. Kilonzo M. N. Yamanis T. J. Singh B. Kajula L. J. (2020). Results from a cluster-randomized trial to evaluate a microfinance and peer health leadership intervention to prevent HIV and intimate partner violence among social networks of Tanzanian men. PloS One, 15(3), Article e0230371. 10.1371/journal.pone.0230371 PMC7083321 32196514

[bibr40-10778012231183660] Marshall K. J. Fowler D. N. Walters M. L. Doreson A. B. (2018). Interventions that address intimate partner violence and HIV among women: A systematic review. AIDS and Behavior, 22(10), 3244–3263. 10.1007/s10461-017-2020-2 29313192 PMC6035885

[bibr41-10778012231183660] Miller E. Decker M. R. McCauley H. L. Tancredi D. J. Levenson R. R. Waldman J. Schoenwald P. Silverman J. G. (2016). A family planning clinic partner violence intervention to reduce risk associated with reproductive coercion: A cluster randomized controlled trial. Contraception, 83(3), 274–280. 10.1016/j.contraception.2010.07.013 PMC3052939 21310291

[bibr42-10778012231183660] Murray L. K. Kane J. C. Glass N. Skavenski van Wyk S. Melendez F. Paul R. Kmett Danielson C. Murray S. M. Mayeya J. Simenda F. Bolton P. (2020). Effectiveness of the Common Elements Treatment Approach (CETA) in reducing intimate partner violence and hazardous alcohol use in Zambia (VATU): A randomized controlled trial. PloS Medicine, 17(4), Article e1003056. 10.1371/journal.pmed.1003056 PMC7164585 32302308

[bibr43-10778012231183660] O'Doherty L. Hegarty K. Ramsay J. Davidson L. L. Feder G. Taft A. (2015). Screening women for intimate partner violence in healthcare settings. Cochrane Database of Systematic Reviews, 2015(7), Article CD007007. 10.1002/14651858.CD007007.pub3 PMC6599831 26200817

[bibr44-10778012231183660] Page M. J. McKenzie J. E. Bossuyt P. M. Boutron I. Hoffmann T. C. Mulrow C. D. Shamseer L. Tetzlaff J. M. Akl E. A. Brennan S. E. Chou R. Glanville J. Grimshaw J. M. Hróbjartsson A. Lalu M. M. Li T. Loder E. W. Mayo-Wilson E. McDonald S. , … Moher D. (2021). The PRISMA 2020 statement: An updated guideline for reporting systematic reviews. British Medical Journal, 372, Article n71. 10.1136/bmj.n71 PMC8005924 33782057

[bibr45-10778012231183660] Piolanti A. Foran H. M. (2022). Efficacy of interventions to prevent physical and sexual dating violence among adolescents: A systematic review and meta-analysis. JAMA Pediatrics, 176(2), 142–149. 10.1001/jamapediatrics.2021.4829 34842911 PMC8630665

[bibr46-10778012231183660] Rhodes K. V. Rodgers M. Sommers M. Hanlon A. Chittams J. Doyle A. Datner E. Crits-Christoph P. (2015). Brief motivational intervention for intimate partner violence and heavy drinking in the emergency department: A randomized clinical trial. Journal of the American Medical Association, 314(5), 466–477. 10.1001/jama.2015.8369 26241598 PMC5637389

[bibr47-10778012231183660] Salazar L. F. Vivolo-Kantor A. Hardin J. Berkowitz A. (2014). A web-based sexual violence bystander intervention for male college students: Randomized controlled trial. Journal of Medical Internet Research, 16(9), Article e203. 10.2196/jmir.3426 PMC4180355 25198417

[bibr48-10778012231183660] Sardinha L. Maheu-Giroux M. Stöckl H. Meyer S. R. García-Moreno C. (2022). Global, regional, and national prevalence estimates of physical or sexual, or both, intimate partner violence against women in 2018. Lancet, 399(10327), 803–813. 10.1016/S0140-6736(21)02664-7 35182472 PMC8885817

[bibr49-10778012231183660] Settergren S. K. Mujaya S. Rida W. Kajula L. J. Kamugisha H. Kilonzo Mbwambo J. Kisanga F. Mizinduko M. M. Dunbar M. S. Mwandalima I. Wazee H. Prieto D. Mullick S. Erie J. Castor D. (2018). Cluster randomized trial of comprehensive gender-based violence programming delivered through the HIV/AIDS program platform in Mbeya Region, Tanzania: Tathmini GBV study. PloS One, 13(12), Article e0206074. 10.1371/journal.pone.0206074 PMC6283609 30521530

[bibr50-10778012231183660] Sharma V. Leight J. Verani F. Tewolde S. Deyessa N. (2020). Effectiveness of a culturally appropriate intervention to prevent intimate partner violence and HIV transmission among men, women, and couples in rural Ethiopia: Findings from a cluster-randomized controlled trial. PloS Medicine, 17(8), Article e1003274. 10.1371/journal.pmed.1003274 PMC7433859 32810146

[bibr51-10778012231183660] Starrs A. M. Ezeh A. C. Barker G. Basu A. Bertrand J. T. Blum R. Coll-Seck A. M. Grover A. Laski L. Roa M. Sathar Z. A. Say L. Serour G. I. Singh S. Stenberg K. Temmerman M. Biddlecom A. Popinchalk A. Summers C. Ashford L. S. (2018). Accelerate progress-sexual and reproductive health and rights for all: Report of the Guttmacher–Lancet Commission. Lancet, 391(10140), 2642–2692. 10.1016/S0140-6736(18)30293-9 29753597

[bibr52-10778012231183660] Stephens-Lewis D. Johnson A. Huntley A. Gilchrist E. McMurran M. Henderson J. Feder G. Howard L. M. Gilchrist G. (2021). Interventions to reduce intimate partner violence perpetration by men who use substances: A systematic review and meta-analysis of efficacy. Trauma, Violence and Abuse, 22(5), 1262–1278. 10.1177/1524838019882357 PMC8649458 31711372

[bibr53-10778012231183660] Sterne J. A. C. Savović J. Page M. J. Elbers R. G. Blencowe N. S. Boutron I. Cates C. J. Cheng H.-Y. Corbett M. S. Eldridge S. M. Emberson J. R. Hernán M. A. Hopewell S. Hróbjartsson A. Junqueira D. R. Jüni P. Kirkham J. J. Lasserson T. Li T. … Higgins J. P. T. (2019). RoB 2: A revised tool for assessing risk of bias in randomised trials. British Medical Journal, 366, Article l4898. 10.1136/bmj.l4898 31462531

[bibr54-10778012231183660] Straus M. A. Hamby S. L. Boney-McCoy S. Sugarman D. B. (1996). The revised conflict tactics scales (CTS2): Development and preliminary psychometric data. Journal of Family Issues, 17, 283–316. 10.1177/019251396017003001

[bibr55-10778012231183660] Taft A. J. Small R. Hegarty K. L. Watson L. F. Gold L. Lumley J. A. (2011). Mothers’ AdvocateS In the Community (MOSAIC)–non-professional mentor support to reduce intimate partner violence and depression in mothers: A cluster randomised trial in primary care. BMC Public Health, 11, Article 178. 10.1186/1471-2458-11-178 21429226 PMC3074545

[bibr56-10778012231183660] United Nations. (2015). *Transforming our world: The 2030 agenda for sustainable development. Resolution adopted by the General Assembly on 25 September 2015 (A/RES/70/1)*.

[bibr57-10778012231183660] Vaillant J. Koussoubé E. Roth D. Pierotti R. Hossain M. Falb K. L. (2020). Engaging men to transform inequitable gender attitudes and prevent intimate partner violence: A cluster randomised controlled trial in North and South Kivu, Democratic Republic of Congo. BMJ Global Health, 5(5), Article e002223. 10.1136/bmjgh-2019-002223 PMC7259847 32467354

[bibr58-10778012231183660] Van Parys A.-S. Deschepper E. Roelens K. Temmerman M. Verstraelen H. (2017). The impact of a referral card-based intervention on intimate partner violence, psychosocial health, help-seeking and safety behaviour during pregnancy and postpartum: A randomized controlled trial. BMC Pregnancy and Childbirth, 17(1), 346. 10.1186/s12884-017-1519-x 28985722 PMC6389099

[bibr59-10778012231183660] Van Parys A.-S. Verhamme A. Temmerman M. Verstraelen H. (2014). Intimate partner violence and pregnancy: A systematic review of interventions. PLoS One, 9(1), Article e85084. 10.1371/journal.pone.0085084 PMC3901658 24482679

[bibr60-10778012231183660] Viechtbauer W. (2005). Bias and efficiency of meta-analytic variance estimators in the random-effects model. Journal of Educational and Behavioral Statistics, 30(3), 261–293. 10.3102/10769986030003261

[bibr61-10778012231183660] Viechtbauer W. (2010). Conducting meta-analyses in R with the metafor package. Journal of Statistical Software, 36(3), 1–48. 10.18637/jss.v036.i03

[bibr62-10778012231183660] Wagman J. A. Gray R. H. Campbell J. C. Thoma M. Ndyanabo A. Ssekasanvu J. Nalugoda F. Kagaayi J. Nakigozi G. Serwadda D. Brahmbhatt H. (2015). Effectiveness of an integrated intimate partner violence and HIV prevention intervention in Rakai, Uganda: Analysis of an intervention in an existing cluster randomised cohort. Lancet Global Health, 3(1), e23–e33. 10.1016/S2214-109X(14)70344-4 PMC4370228 25539966

[bibr63-10778012231183660] World Health Organization/London School of Hygiene and Tropical Medicine (2010). Preventing intimate partner and sexual violence against women: Taking action and generating evidence. World Health Organization.

[bibr64-10778012231183660] Zhang J. Yu K. F. (1998). What’s the relative risk? A method of correcting the odds ratio in cohort studies of common outcomes. Journal of the American Medical Association, 280(19), 1690–1691. 10.1001/jama.280.19.1690 9832001

